# Consumer acceptance of egg white partially substituted with mushrooms and mushroom–egg white flavor pairing

**DOI:** 10.1002/fsn3.2105

**Published:** 2021-01-28

**Authors:** Xiaofen Du, Adriana Muniz, Joanna Sissons, Wanyi Wang, Shanil Juma

**Affiliations:** ^1^ Department of Nutrition and Food Sciences Texas Woman’s University Denton TX USA; ^2^ Center for Research Design & Analysis Texas Woman’s University Houston TX USA

**Keywords:** *Agaricusbisporus*, consumer test, crimini, egg white patties, flavor pairing, white mushroom

## Abstract

Mushroom possesses a distinctive sensory quality and unique nutrients. Its pairing with egg white and consumer acceptance has never been investigated. In this study, formulated mushroom–egg white patty prototypes (white and crimini mushrooms at 0%, 10%, 20%, and 30%, either oven roasted or steamed) were evaluated by 380 participants for acceptance and intensity of nine sensory attributes. Mushroom–egg white patty prototypes received positive hedonic scores for overall acceptance and the likeability of overall flavor, mushroom flavor, meaty flavor, egg white flavor, overall texture, and firmness. Consumer overall acceptance was most strongly and positively correlated with overall flavor liking, followed by overall appearance and overall texture likeability. Additionally, the likeability of flavor pairing between mushroom and egg white was rated positively across all 16 patties, indicating a good flavor match of mushroom and egg white. Consumer hedonic levels toward mushroom patties were significantly (*p* ≤ .05) impacted by cooking method, mushroom type, and mushroom level. The addition of mushroom was acceptable up to 20%, with steam method and crimini mushroom most preferred. The results provided new insights into consumer attitudes and potentially important sensory factors affecting the acceptability of mushroom–egg white patties, consequently increasing the utilization and consumption of mushrooms and mushroom‐blended products.

## Graphical abstract

Mushroom–egg white patty consumer acceptance was mainly driven by overall flavor likeability. Mushroom and egg white had a good flavor matching. Up to 20% mushroom addition was an acceptable supplement to egg white patties. Cooking method and mushroom proportion had significant impact on hedonic rating.

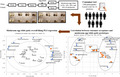

## 1 INTRODUCTION

The global mushroom industry is currently valued at approximately $70 billion (Grimm et al., 2020). More than half of the cultivated mushrooms are freshly consumed, and the rest are processed into products such as dried, canned, fresh‐cut, frozen, or powdered and used as food ingredients such as seasoning in soups, sauces, noodles, and bakery products (Salehi, 2019; Siddiq et al., 2018). Possessing a distinctive aroma, taste, texture sensory quality, and unique health‐promoting and disease‐preventing dietary components makes mushroom an ideal food supplement. Mushrooms are low in calories, fat, sodium, and cholesterol, but high in protein, carbohydrates, dietary fiber, and micronutrients (Kalač, 2016a). Nevertheless, the global mushroom consumption is approximately 4.5 kg annually per capita (Robinson et al., 2019). There is ample room to increase incorporating mushrooms in dishes to promote consumption.

Egg white (albumin) is a widely consumed animal protein with a well‐balanced amino acid composition. Approximately 6.9 billion hens were responsible for producing 1,250 billion eggs for a world population of 7.13 billion people (Bertechini, 2017). There is a consensus across science, industry, and government that increasing the proportion of plant‐based foods in diets will have benefits on environmental sustainable, public health, and animal welfare (Lang, 2020). Pure egg white possesses a quite bland flavor, but can serve various food functions such as emulsifier, aerator, thickener, and binder (Mine, 2015). Egg white is widely used as a binder for plant‐based meat substitutes, contributing binding and texture during consumption (Kumar et al., 2017). In contrast, mushrooms have distinguished flavor properties, attributing primarily to water‐soluble substances such as 5′‐nucleotides, free amino acids, and soluble carbohydrates, which are associated with umami and sweet tastes and can enhance meat‐like flavor (Kalač, 2016a; Poojary et al., 2017; Zhang et al., 2013). In addition, approximately 150 volatiles have been identified in various mushrooms, with C_8_ aliphatic volatiles contributing to mushroom aroma (Aisala et al., 2020; Combet et al., 2006; Grosshauser & Schieberle, 2013; Zhou et al., 2015). Nevertheless, no study has investigated dishes blending with mushroom and egg white.

Limited studies have focused on consumer acceptability of raw or cooked mushrooms (Aisala et al., 2020; Barroso et al., 2019). The focus of such research has been consumer acceptance of different dishes or patties with mushrooms added. For example, consumer tests involving the addition of mushroom to savory products such as sausage‐type, beef patties, or chicken patties (Akesowan, 2016; Guinard et al., 2016; Spencer & Guinard, 2018; Stephan et al., 2018; Tom et al., 2018; Wong et al., 2017, 2019). Additionally, several studies have evaluated different mushroom powders applied to bakery products and their consumer acceptance (Salehi, 2019). These studies have demonstrated the application of mushrooms as part of food patties interests the scientific community.

The blending of mushroom and egg white in consumer‐friendly products can be synergistic in their sensory properties, nutritional values, and health benefits. Nevertheless, to the best of our knowledge, no studies have focused on consumers’ acceptance and preference for mushroom–egg white blends. This study aimed to develop prototypes of mushroom–egg white patties, investigate their consumer acceptance, and identify mushroom–egg white flavor pairing qualities. Two *Agaricus bisporus* mushrooms (white and crimini) were used in this study, since they are the most commonly consumed mushroom species (Robinson et al., 2019). The most common consumption scenario is the culinary use including roasted mushrooms in salads, entrees, burgers, and sandwiches (Du et al., 2021).

## 2 MATERIALS AND METHODS

### 2.1 Mushroom–egg white patty experimental design

White and crimini mushrooms (*Agaricus bisporus*) were freshly picked and sent to Texas Woman’s University the next morning by a local mushroom supplier, J‐M Farms, Inc. (Miami, OK, USA). The fresh mushrooms were saved in a refrigerator (4°C) and used within three days. Egg white was purchased from MuscleEgg (Spanish Fork, UT, USA). The egg white was pure, frozen liquid without any food additives and was frozen (−20°C) until used.

A total of 16 patties were developed, and the composition of the patties is shown in Table 1. The design included three factors: mushroom types (white and crimini), mushroom levels (0%, 10%, 20%, and 30% by weight after roasting in the final samples), and cooking methods (steamed and roasted). The 10%, 20%, and 30% mushroom proportions were selected for this study, as the mushroom levels beyond 30% caused huge deviations from appearance and uniformity and binding ability with egg white. The patty development was based on trial and error with the input from an experienced restaurant chief.

**TABLE 1:** Mushroom–egg white patty sample information and consumer test design. Number of total participants *n* = 380

Mushroom species	Used percent	Cooking methods	Panel size	Sensory attributes	Scales
White (*Agaricus bisporus*)	0, 10%, 20%, 30%	Roasted	*n* = 92	Overall liking Overall appearance liking Overall flavor liking Overall texture liking Mushroom flavor liking Mushroom flavor intensity Meaty flavor liking Meaty flavor intensity Egg white flavor liking Egg white flavor intensity Firmness texture liking Firmness texture intensity Mushroom–egg white flavor pairing liking Mushroom–egg white flavor pairing intensity	9‐point hedonic scale for nine liking questions: 1 = dislike extremely 2 = dislike very much 3 = dislike moderately 4 = dislike slightly 5 = either like or dislike 6 = like slightly 7 = like moderately 8 = like very much 9 = like extremely 9‐point line scale for five intensity questions: 0 = none 5 = moderate 9 = extremely strong
White (*Agaricus bisporus*)	0, 10%, 20%, 30%	Steamed	*n* = 100		
Crimini (*Agaricus bisporus*)	0, 10%, 20%, 30%	Roasted	*n* = 91		
Crimini (*Agaricus bisporus*)	0, 10%, 20%, 30%	Steamed	*n* = 97		

### 2.2 Roasted mushroom preparation

Similar to our previous study (Du, Sissons, Shanks, & Plotto., 2021), mushrooms (white and crimini) were rinsed in tap water. A soft bristle brush was used to remove residue, if needed. The cleaning procedure lasted for 5–10 s to avoid excess water retention in mushrooms. Mushrooms were then drained in a steel strainer sieve metal bowl with paper towel to fully dry. The stipe in mushrooms was removed by a knife and discarded. The mushroom caps were diced into ¼ in cubes using a mandolin slicer (Fullstar Houseware LLC, New York, NY, USA). The diced mushrooms were added to a stainless steel mixing bowl sprayed with a small amount of canola oil and gently mixed with a large rubber spatula. The ratio of oil:diced mushrooms was approximately 1:200. Any excessive oil was wiped out with a paper towel. A sheet pan was evenly wetted with canola oil, and the diced mushrooms were spread in a single layer evenly. The pan was then placed in a preheated (163°C) convection oven (Blodgett SHO‐100‐G Natural Gas Single Deck Full Size, Essex Junction, VT, USA) under low fan settings.

Oven temperature was controlled precisely and checked using an oven thermometer. When mushrooms were roasted for 2–3 min, small droplets of water developed and the sheet pans were quickly removed from the oven. The diced mushrooms were turned using a small rubber spatula and spread evenly on the sheet pan again. Then, the sheet pan was returned to the oven to finish cooking. Mushrooms were roasted for approximately 8 min total. Mushrooms were done when much of the water had evaporated and browning had just begun. Moisture loss due to cooking was approximately 50% by weight. After done, the roasted mushroom dices were quickly transferred to a holding vessel. Kosher salt was spread with a ratio of salt to mushroom dices at 0.25 g salt/100 g roasted mushrooms.

### 2.3 Mushroom–egg white patty preparation

Liquid raw egg white was removed from the freezer, allowed to reach room temperature (15–21°C), and used within 2 hr. Muffin pans (silicone and metal) were slightly sprayed with canola oil, and the excess oil was wiped away. Liquid egg white was measured and added into muffin pans with a 10‐ml pipette. The volumes of egg white were 10 ml (control without mushroom), 9, 8, and 7 ml. The cooled roasted mushroom dices were weighed at 1, 2, and 3 g, respectively, and mixed with 9, 8, or 7 ml egg white in muffin pans using a glass stir bar.

Two cooking methods for mushroom–egg white patties were applied: steam and roast. For steamed mushroom–egg white patties, the silicone muffin pans were steamed at 163°C for approximately 10 min on the stove (Vulcan SX60‐10BN SX, Baltimore, MD, USA). For roasted mushroom–egg white blend, the Blodgett convection oven was preheated to 163°C with low fan. The metal muffin pans were placed into the convection oven and roasted at 163°C for 9 min. An oven thermometer was used to accurately monitor temperature. Cook time varied slightly based on volume of samples, and the patties were done when set firm. Moisture loss for steamed mushroom–egg white patties was approximately 5%, while for roasted patties was approximately 25%. After cooking, the small amount of water on the top of the patties was blotted with a paper towel. Then, the mushroom patties were removed from muffin pans using a rubber spatula, transferred to a holding vessel, and placed in the serving cups (60‐ml plastic cups with lids, AV Inc., Brooklyn, NY, USA), one patty per cup, and stored in an insulated food carrier (Cambro EPP400110 Cambro^®^, Huntington Beach, CA, USA). A tray with one gallon boiled water (~90°C) was set on the bottom of the carrier, which could hold the temperature up to 3 hr at 50–60°C. Multiple patties for each mushroom type, proportion, and cooking method were prepared for the consumer tests.

### 2.4 Consumer tests

All sensory procedures were reviewed and approved by the Texas Woman’s University (TWU) Institutional Review Board (IRB). Participants were recruited primarily from TWU (Denton, Texas) through bulk emails sent to students, faculty, and staff. Participants were preselected based on their frequency of mushroom consumption and nonallergies to egg. Once selected, eligible participants were notified via email and scheduled to participate in 1–4 sessions (Table 1; 16 samples were divided into four sessions with four patties in each session). A total of 380 voluntary participants attended in this study (Table 1).

Upon arrival at the TWU sensory laboratory, participants were signed‐in, read the consent form, and signed and dated the consent form before proceeding. A trained researcher escorted participants into a partitioned sensory booth illuminated with incandescent lighting to discuss the sensory booth setup and testing procedures. Participants were encouraged to ask questions before starting the sample sensory evaluation.

For each taste session, participants received a tray with four patties in four portion cups, one 354‐ml cup of drinking water (Nestle Pure Life Water, Stamford, CT, USA), two piece of unsalted Saltine crackers (Nabisco’s Premium, East Hanover, NJ, USA), one piece of napkin, one stapled test ballot, and a pencil. All patties were served in 60‐ml plastic portion cups covered with a plastic lid, and the serving size for each sample was one patty (7–10 g). The cup was identified with a randomized three‐digit code. The order of presentation of the patty samples was randomized for each participant to minimize bias. The test ballot included instructions and a score sheet. Participants were instructed to rinse their mouth with water first, sample one patty at a time, take a bite of cracker, and swish their mouth with water before sampling the next patty.

Participants rated their degree of acceptance for each patty, including 14 sensory attributes (Table 1). A 9‐point hedonic scale was used for evaluating four overall impression and five attributes liking, while a 9‐point line scale was used to evaluate five attribute intensities. The rating intensity was based on an unstructured 0–9 line scale with 0 at the left end, 9 at the right end, and 5 in the middle. The sensory test session for each panelist was approximately 30 min, although the participants were encouraged to take their time. After evaluation, participants completed an exit survey including seven demographic questions related to age, gender, mushroom consumption frequency, mushroom consumption form, fresh mushroom preparation methods, egg consumption frequency, and diet pattern. Each participant received a cash honorarium for participation.

### 2.5 Statistical analysis

Repeated measures multivariate analysis of variance (MANOVA) was performed on ratings of 14 attributes across all 16 mushroom–egg white patties (2 cooking methods × 2 types of mushroom × 4 mushroom levels). Once significant levels were identified, univariate analysis of variance (ANOVA) was used to examine the effect of each factor on liking and intensities. If interaction or main effects were found, least significant difference (LSD) test was performed for the pairwise comparisons with *p*‐value ≤ .05. Pearson correlation coefficients were calculated to determine the relationship between attribute liking and intensities. Principal component analysis (PCA) was used to assess the similarities and differences among the mushroom patties using the covariance matrix with attribute liking and intensity ratings as loading values. Partial least squares (PLS) was conducted to identify attributes projected to overall liking of the mushroom patties. Hierarchical cluster analysis (HCA) was conducted for examining consumer segments of demographic data. Statistical analyses were performed using SPSS version 25 (IBM SPSS Statistics, Armonk, NY, USA) and XLSTAT 2019 (Addinsoft, New York, NY, USA).

## 3 RESULTS

### 3.1 Mushroom–egg white patty hedonic rating

The overall acceptability of the 16 mushroom patties ranged from 4.7 to 6.7 on a 9‐point hedonic scale (5 meant either like or dislike), implying the formulated patties were acceptable commonly (Table 2). Overall acceptability of the eight steamed mushroom patties with 0%–30% white and crimini mushrooms, respectively, had a mean rating above 5. No significant (*p* ≤ .05) difference was identified across these eight patties. Overall acceptability of the roasted patties with mushroom proportions up to 20% did not show significant (*p* ≤ .05) difference from the control patties (with 0% mushroom). Only the roasted patties with 30% white mushroom scored below 5, which was significantly (*p* ≤ .05) different in comparison with other roasted patties.

**TABLE 2:** Mean scores of nine liking attributes for the consumer study of 16 mushroom–egg white patties

			Overall liking	Overall appearance liking	Overall flavor liking	Overall texture liking	Mushroom flavor liking	Meaty flavor liking	Egg white flavor liking	Firm texture liking	Mushroom‐egg white flavor pairing liking
Steamed	White	0%	6.06a	6.70b	6.24a	6.52b	‐	‐	6.64b	6.48b	‐
		10%	5.92a	6.00b	6.05a	6.15b	5.79a	5.53a	6.00a,b	6.06a,b	5.90a
		20%	5.92a	5.24a	6.30a	5.72a,b	6.41a	5.79a	6.10a,b	5.67a,b	6.15a
		30%	5.55a	4.64a	5.89a	5.13a	6.07a	5.78a	5.74a	5.37a	5.66a
	Crimini	0%	6.16a	6.52b	6.04a	6.57b	‐	‐	6.53b	6.58b	‐
		10%	6.30a	5.91b	6.29a	6.11a,b	6.50a	5.60a	6.36a,b	5.95a,b	6.35a
		20%	6.17a	5.07a	6.04a	5.79a,b	6.49a	5.98a	5.91a,b	5.86a,b	6.19a,b
		30%	5.81a	4.95a	6.04a	5.33a	6.24a	5.87a	5.67a	5.44a	5.59a
Roasted	White	0%	5.99b	6.22b	6.05b	5.90b	‐	‐	6.53b	5.85b	‐
		10%	5.22a,b	4.05a	5.66a,b	5.03a,b	5.85a	5.78a	5.77a,b	5.07a,b	5.53a
		20%	5.16a,b	3.49a	5.71a,b	4.66a	5.98a	5.93a	5.84a,b	5.28a,b	5.60a
		30%	4.72a	3.50a	5.10a	4.17a	5.16a	5.47a	5.27a	4.73a	4.98a
	Crimini	0%	6.67b	7.15c	6.70b	6.79c	‐	‐	6.99c	6.68c	‐
		10%	5.70a	4.75b	6.03a,b	5.89b	6.24a	5.82a	6.40b,c	6.09b,c	5.99a
		20%	5.80a,b	3.90a,b	6.19a,b	5.48a,b	6.37a	6.15a	6.05a,b	5.68a,b	6.01a
		30%	5.24a	3.52a	5.52a	4.90a	5.61a	5.60a	5.68a	5.18a	5.30a
Cooking method		Steamed	5.99b	5.64b	6.11b	5.92b	6.25b	5.76a	6.13a	5.93b	5.99b
		Roasted	5.55a	4.58a	5.88a	5.36a	5.88a	5.80a	6.07a	5.58a	5.58a
Mushroom type		White	5.59a	5.01a	5.88a	5.43a	5.88a	5.71a	5.99a	5.58a	5.65a
		Crimini	6.00b	5.25b	6.12b	5.88b	6.27b	5.84a	6.21b	5.95b	5.94b
Mushroom level		0%	6.20c	6.64c	6.25b	6.45c	‐	‐	6.67c	6.40c	‐
		10%	5.80b,c	5.21b	6.02a,b	5.81b	6.10a,b	5.68a	6.14b	5.81b	5.95b
		20%	5.78b	4.44a	6.07b	5.42b	6.32b	5.96a	5.98b	5.63b	6.00b
		30%	5.32a	4.17a	5.64a	4.87a	5.77a	5.68a	5.59a	5.18a	5.39a
*F*‐value	Cooking method (C)		13.60***	91.16***	4.47*	23.47***	7.95**	0.08	0.30	9.25**	8.80**
	Mushroom type (M)		12.89***	4.72*	4.11*	14.90***	7.29**	1.32	4.56*	10.10**	3.87*
	Mushroom level (L)		9.63***	101.64***	5.00**	31.69***	5.30**	3.01	19.74***	18.34***	7.86***
	C*M		2.07	6.13*	4.78*	10.65**	0.11	0.01	4.66*	6.91**	0.89
	C*L		3.55	12.15	2.56	1.12	2.11	2.17	0.70	0.16	0.08
	M*L		0.02	0.30	0.15	0.02	0.51	0.17	1.07	0.21	0.50
	C*M*L		0.21	1.76	0.58	0.12	0.61	0.01	0.10	0.78	0.21

Note

Different letters within each attribute across different mushroom proportions indicate significant difference between samples (one‐way ANOVA, *p *≤ .05). F‐values for cooking method (C), mushroom type (M), and mushroom level (L) main effects, as well as interactions between them indicate their impact on each attribute (three‐way ANOVA, *p *≤ .05).

*, **, ***: significant at *p *< .05, *p *< .01, and *p *< .001, respectively.

Appearance is the first parameter that participants perceive food, and it plays a key role in consumer product evaluation and choice. The overall appearance acceptability of the 16 patties ranged from 3.5 to 7.2 (Table 2). Steamed patties with mushroom proportions up to 20% had appearance hedonic scores higher than 5, while all roasted patties (10%–30% mushrooms) were rated below 5, indicating non‐favorite appearance for the roasted method.

The overall flavor of all mushroom patties was acceptable, ranged from 5.1 to 6.6 for all 16 patties (Table 2). All eight steamed mushroom patties did not show significant (*p* ≤ .05) difference for overall flavor acceptability, while the roasted patties with mushroom proportions up to 20% did not show significant (*p* ≤ .05) difference from the control. The flavor of mushroom–egg white patties was primarily imparted by two major ingredients: mushroom and egg white. The mushroom flavor acceptability for the 12 patties ranged from 5.2 to 6.5, indicating that participants liked the mushroom flavor commonly. It is well known that mushroom flavor has association with meat‐like flavor (Poojary et al., 2017). Meaty flavor acceptability for all mushroom patties was above 5 without significant (*p* ≤ .05) difference across samples, inferring that consumer liked the meaty flavor of the patties. The egg white flavor acceptability of the 16 patties ranged from 5.3 to 7.0, showing egg white flavor was favored by participants (Table 2). Both steamed and roasted patties with mushroom proportions up to 20% did not show significant (*p* ≤ .05) difference for egg white flavor acceptability across samples.

The overall texture was acceptable in general, with ratings ranged from 4.2 to 6.8 for all patties (Table 2). Only the roasted patties with 20% and 30% white mushroom, respectively, had average scores below 5, while steamed mushroom patties with white and crimini proportions up to 20% did not show significant (*p* ≤ .05) difference from the control. Firmness was part of the overall texture. Firmness acceptability for the 16 patties ranged from 4.7 to 6.7. Only the roasted patty with 30% white mushrooms was rated below 5, while the remaining patties had an average score above 5.

### 3.2 Mushroom–egg white patty flavor intensity

The intensities of firm texture, mushroom flavor, meaty flavor, and egg white flavor ranged 4.4–6.3, 4.5–7.3, 3.9–6.4, and 2.5–7.7 out of a 9‐point line scale, respectively (Table 3). The variance of the intensities of these four sensory attributes across all patties depended on the cooking method, the ratio between mushroom and egg white, and the mushroom type. Mushroom proportion up to 20% showed significantly (*p* ≤ .05) higher intensities of these attributes, compared to 10% mushroom patties and the control. Pearson correlation analysis (data not shown) indicated that the intensities of mushroom flavor, meaty flavor, and egg white flavor were highly, positively correlated with the likeability of mushroom flavor, meaty flavor, and egg white flavor, with *r* = .93, .96, and .89, respectively. It implied that more the intense of these three flavors, the higher likeability. Firmness intensity was negatively correlated with firmness acceptability (*r* = −.82), implying more firmness, less likeability. It has been observed that more mushroom in the patties, harder texture, consequently caused less acceptance (Tables 2 and 3).

**TABLE 3:** Mean scores of five intensity attributes for the consumer study of 16 mushroom–egg white patties

			Mushroom flavor intensity	Meaty flavor intensity	Egg white flavor intensity	Firm texture intensity	Mushroom‐egg white flavor pairing intensity
Steamed	White	0%	‐	‐	7.23d	4.95a	‐
		10%	4.46a	3.89a	5.97c	5.28a	5.49c
		20%	6.67b	4.99b	4.85b	5.27a	4.41b
		30%	7.08b	5.70b	3.56a	6.20b	3.58a
	Crimini	0%	‐	‐	6.88d	4.48a,b	‐
		10%	4.73a	3.89a	5.68c	4.43a	5.42c
		20%	6.74b	5.25b	4.14b	5.37b,c	4.27b
		30%	7.28b	5.53b	3.09a	5.65c	3.24a
Roasted	White	0%	‐	‐	7.72d	5.37a	‐
		10%	4.67a	4.31a	5.29c	5.51a,b	5.17c
		20%	6.64b	5.88b	4.10b	6.06a,b	3.93b
		30%	7.09b	6.40b	3.00a	6.26b	3.05a
	Crimini	0%	‐	‐	7.68d	5.17a	‐
		10%	4.79a	4.57a	5.26c	5.16a	5.26c
		20%	6.83b	5.82b	3.57b	5.68a,b	4.01b
		30%	7.29b	5.78b	2.50a	6.16b	3.04a
Cooking method		Steamed	6.13a	4.86a	5.21b	5.20a	4.43b
		Roasted	6.19a	5.45a	4.93a	5.66b	4.11a
Mushroom type		White	6.10a	5.18a	5.22b	5.61b	4.28a
		Crimini	6.21a	5.10a	4.92a	5.24a	4.28a
Mushroom level		0%	‐	‐	7.37a	4.99a	‐
		10%	4.66a	4.16a	5.56b	5.09a	5.34c
		20%	6.72b	5.47b	4.17c	5.59b	4.16b
		30%	7.18c	5.86c	3.07d	6.08c	3.24a
*F*‐value	Cooking method (C)		0.25	20.34***	6.96**	16.49***	8.83**
	Mushroom type (M)		2.29	0.20	11.46**	8.99**	0.37
	Mushroom level (L)		179.00***	63.24***	293.74***	17.67***	122.83***
	C*M		0.00	0.46	0.72	0.65	1.16
	C*L		0.13	0.35	8.46***	0.29	0.16
	M*L		0.05	1.72	1.09	0.68	0.28
	C*M*L		0.12	0.73	0.12	0.99	0.06

Different letters within each attribute across different mushroom proportions indicate significant difference between samples (one‐way ANOVA, *p *≤ .05). F‐values for cooking method (C), mushroom type (M), and mushroom level (L) main effects, as well as interactions between them indicate impact on each attribute (three‐way ANOVA, *p *≤ .05). *, **, ***: significant at *p *< .05, *p *< .01, and *p *< .001, respectively.

### 3.3 PCA of mushroom patties and PLS overall liking projected to sensory attributes

**FIGURE 1:**
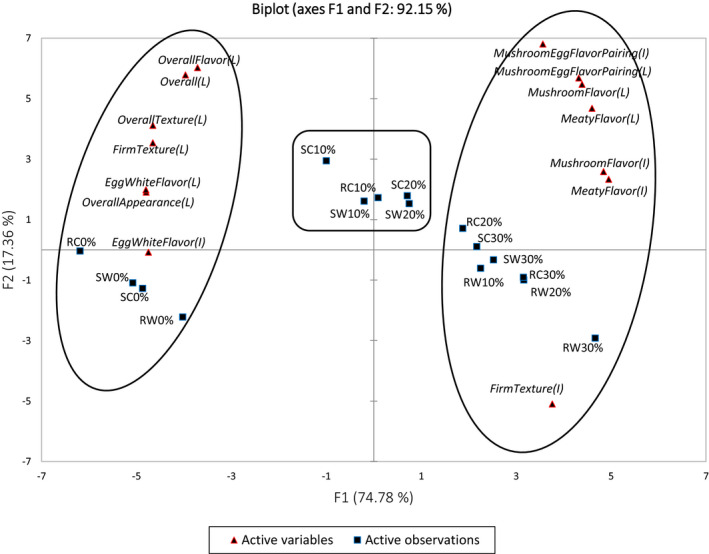
Principal component analysis (PCA) biplot of mean scores of 14 attribute ratings across 16 mushroom–egg white patties. C, Crimini mushroom; I, Intensity; L, liking; R, Roasted; S, Steamed; W, White mushroom. According to XLSTAT PCA output of “Squared cosines of the variables” and “Squared cosines of the observations,” two big oval circles were created based on variables (attributes) and observations (samples) were significantly separated from other samples either at positive or negative side of PC1. The rectangle was created based on variables (attributes) and observations (samples) were significantly separated from other samples at positive side of PC2

Principal components analysis was performed to visualize the underlying relationships between 14 sensory attributes (loadings) and 16 mushroom–egg white patties (scores) (Figure 1). The first two PCs accounted for 92.15% of the total variance. The PC1 axis explained 74.78% of the variance alone, which was the major component to differentiate samples by their sensory attributes. Four control samples were separated at the negative side of PC1, possessing high scores in overall acceptability, acceptance in overall appearance, overall texture, firmness, overall flavor, and egg white flavor, as well as egg white flavor intensity. In contrast, eight mushroom patties (steamed and roasted patties with white and crimini mushrooms at 20% and 30%, respectively) were separated at the positive side of PC1, which were characterized with high intensities in firmness, mushroom flavor, meaty flavor, and mushroom–egg white flavor pairing, receiving high scores in the acceptance of mushroom flavor, meaty flavor, and mushroom–egg white flavor pairing. Five mushroom patties, namely steamed patties with 10% and 20% white and crimini mushrooms and roasted patties with 10% crimini, respectively, were separated in the positive side of PC2, which lacked typical high loading scores of sensory attributes in this PC1 vs PC2 biplot. Their hedonic scores, however, were in between the mushroom patties separated on the positive and negative sides of PC1 (Table 2). The PCA biplot clearly showed the distribution of hedonic ratings and intensities of sensory attributes across the 16 mushroom patties.

**FIGURE 2:**
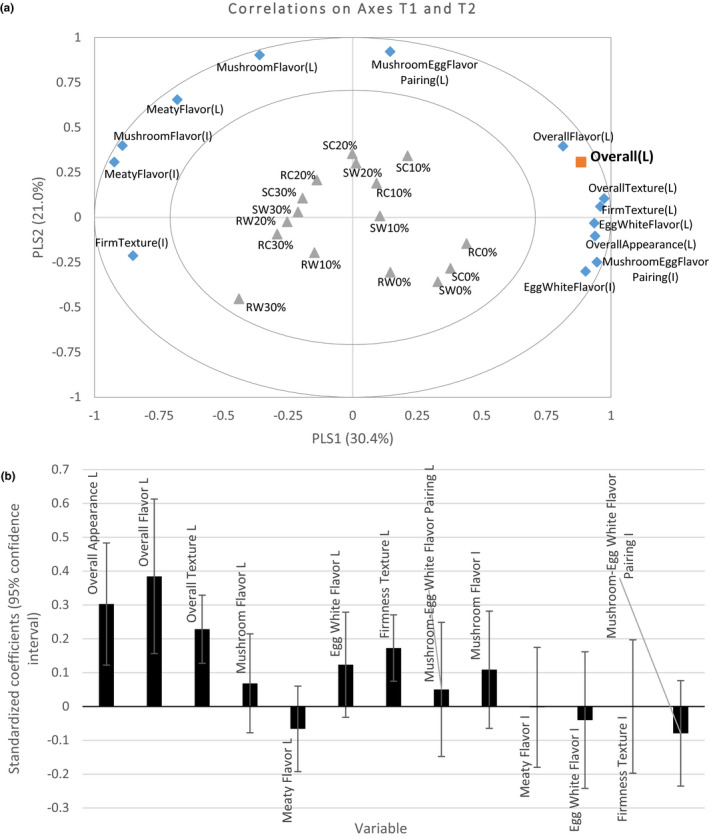
Partial least square (PLS) regression for the associations of overall liking and all rest attribute liking and intensities for 16 mushroom‐egg patties, including (a) PLS biplot with X and Y variables on axes t1 and t2, and (b) standardized coefficients of every attribute to predict overall liking. C, Crimini mushroom; I, Intensity; L, liking; R, Roasted; S, Steamed; W, White mushroom

To investigate the endogenous sensory attribute related to overall acceptability, PLS was performed. As shown in Figure 2a, the first two principal components were found to explain up to 51% of variance, while the predictive model quality was 89% when the three dimensions were retained in the model (PLS output, data not shown). The first two components obtained from the analysis were used for each regression as their *R* ^2^ and *Q* ^2^cum values (*R* ^2^ = 0.94, *Q* ^2^cum = 0.91, and RMSE = 0.102) indicated a good fit model. Overall liking of 16 mushroom patties were highly correlated with 13 sensory attributes. The acceptability of overall flavor, overall appearance, overall texture, firmness texture, egg white flavor, and mushroom flavor intensity were most effectively predicting the overall liking, with Variable Importance in Projection (VIP) > 1.0 (PLS output, data now shown). Seven samples (four patties without mushrooms and three patties with 10% mushrooms) were highly correlated with the overall liking, which was consistent with PCA results. The standardized coefficients with 95% confidence interval of PLS are displayed in Figure 2b, in which the larger coefficient indicates a more important driver. It indicated that overall flavor acceptability was the most significant, impactful factor, followed by the acceptability of overall appearance and overall texture.

### 3.4 Mushroom–egg white flavor pairing

Since flavor is the key driver for overall acceptability, besides mushroom flavor, meaty flavor, and egg white flavor, mushroom–egg white flavor pairing was also investigated. The hedonic scores of mushroom–egg white flavor pairing of the patties ranged from 5.0 to 6.4 (Table 2), implying that the flavor pairing of mushroom and egg white is favored. The cooking method had a significant (*p* ≤ .05) impact on the flavor pairing: Steamed mushroom patties were rated higher than roasted mushroom patties. Mushroom type had a significant (*p* ≤ .05) impact on the flavor pairing, with crimini rating higher than white mushrooms. Mushroom levels also had a significant (*p* ≤ .05) impact on the acceptability of mushroom–egg white flavor pairing. The patties with 10% and 20% mushroom were rated higher than the patties containing 30% mushrooms.

The mushroom–egg white flavor pairing intensity of the 12 mushroom–egg white patties ranged from 3.1 (e.g., roasted 30% white patty) to 5.5 (e.g., steamed 10% white and crimini patties, respectively) (Table 3). Four patties with 10% mushroom had intensity scores ranged 5.2–5.5, implying the flavor balanced well between egg white and mushrooms. When the mushroom proportion increased to 20% and 30%, the flavor pairing intensities shifted to the side of more mushroom flavor. It meant mushroom flavor increased along with the mushroom proportions in the patties, inferring the more mushrooms in the patties, the less egg white flavor perceived. The flavor pairing intensity showed significant (*p* ≤ .05) difference along with cooking methods that steamed patties had more egg white flavor than roasted ones.

### 3.5 Impact of cooking method, mushroom type, and level

The impact of cooking method on mushroom patty prototype acceptance was significant (*p* ≤ .05): The hedonic ratings of all attributes were higher for the steamed mushroom patties than for the roasted patties, regardless of mushroom type and level (Table 2). For example, the steamed mushroom patties were rated significantly higher on overall acceptability the likability of overall appearance, overall texture, firmness, overall flavor, and mushroom flavor, compared to the roasted patties with 10% and 20% mushrooms, respectively. The impact of cooking method on the intensities of mushroom and meaty flavors was insignificant, while it had a significant impact on egg white flavor intensity as well. In contrast, roasted method demonstrated stronger firmness intensity than the steamed ones (Table 3).

Overall acceptability of the mushroom patties also depended upon mushroom type and proportions. Compared to white mushroom patties, crimini mushroom patties were rated significantly (*p* ≤ .05) higher on overall acceptability and the likability of overall appearance, overall texture, firmness, overall flavor, mushroom flavor, and egg white flavor, regardless of mushroom level and cooking method (Table 2). In contrast, white mushroom patties had significantly (*p* ≤ .05) higher intensities in firmness texture and egg white flavor.

The impact of mushroom levels on patty acceptability scores was significant (*p* ≤ .05) (Table 2). Increasing mushrooms in the patties decreased ratings on overall acceptability with the control having the highest scores. The patties containing 30% mushrooms received a significantly lower scores of overall acceptability than those with 0%–20% mushrooms, although the ratings were still above 5. A similar trend was observed for the likability of overall appearance, overall texture, firmness, overall flavor, mushroom flavor, meaty flavor, and egg white flavor, regardless of mushroom types and cooking methods. The 20% mushroom level was a cut‐point. The least overall flavor acceptability of the patties containing 30% mushrooms might be caused by too much mushroom flavor, as higher mushroom concentrations created stronger mushroom and roasted flavors.

The mushroom levels had a significant (*p* ≤ .05) impact on the intensities of mushroom flavor and meaty flavor with patties containing 10% mushroom rated lowest and 30% mushroom rated highest. The impact of the mushroom level on the firmness intensity was significant. The patties with 30% mushroom were rated more firm than the other levels, and patties with 20% mushroom were firmer than 10% and control. It seemed that mushroom levels were positively correlated with flavor intensity, but negatively impacted attribute acceptability. Nevertheless, the relationship was not significant (Pearson correlation analysis, data not shown).

## 4 DISCUSSION

The findings of this study indicated that the overall acceptability of mushroom–egg white patties with mushrooms up to 30% received positive hedonic scores (above the midpoint of the scale). It implied that the sensory properties of formulated patties were quite acceptable. Studies have shown consumer acceptance toward meat products substituted with mushroom: The overall liking of variant beef patties with mushroom substitution up to 30% was similar to the all‐meat control (Wong et al., 2019); variant dishes with mushroom up to 45% had similar overall liking to the all‐meat control (Wong et al., 2017, 2019); and no significant difference in overall liking was observed among six recipes where beef had been partially substituted with mushrooms (Guinard et al., 2016). It is well known that mushrooms have certain “meaty” taste and are suitable to be used as meat analogues (Kumar et al., 2017). The results of this study added knowledge that mushrooms have potential to be blended with egg white to make paired products.

The appearance of the mushroom–egg white patties may need further improvement to increase hedonic scores. The Maillard reaction is the primary mechanism responsible for the darkened appearance. Roasted patties had a darker (browner) appearance compared to the control. Crimini mushroom patties had a darker color than white mushroom patties, while higher mushroom levels caused a darker color. Panelists may perceive the dark color as a negative attribute, and it was important to manage cooking temperature and time. The appearance acceptability in this study was consistent with a report that consumers had significantly less appearance acceptance toward beef that had been partially substituted with mushrooms (Guinard et al., 2016), while controversy to another report that consumers had higher appearance preference toward high mushroom/low meat compared to low mushroom/high meat mixed dishes (Spencer & Guinard, 2018). It indicated that consumer appearance acceptability depended on dishes or applications.

The flavor of the mushroom‐egg white patties was preferred by participants. Flavor is the primary factor determining the acceptability of a product, and it has the highest impact as far as the market success is concerned (Andersen et al., 2019). The C_8_ aliphatic volatiles and a wide range of umami‐inducing nonvolatiles from mushrooms, as well as reacted volatile compounds such as pyrazines, furans, and pyrroles from the Maillard reaction, are associated with the umami, meaty, and savory flavors of mushrooms (Kalač, 2016b). When egg white is cooked, the Maillard reaction created certain heterocyclic nitrogen‐containing compounds, sulfurs, and ketones, which contribute to cooked egg white flavor (Maga, 1982; Umano et al., 1990). The results suggested that a well control of Maillard reaction would highly determine mushroom–egg white patty flavor.

The texture of the mushroom patties was positively accepted by participants. The patty texture was determined by egg white gel formation, which involves a complex process of egg white protein denaturation and aggregation (Mine, 2015). In general, higher temperature and less water‐binding cause a stronger gel, consequently resulting in a firmer texture. On the other hand, reduction in moisture content in roasted mushrooms causing an increase in fiber content may affect water absorption and moisture content. Major fiber in mushroom is chitin and beta‐glucans, which account up to 4%–20% in dry weight basis (Zhu et al., 2015). This can result in a more compact structure with higher density and a more firm texture. Therefore, more mushroom in the patties can influence texture properties. The 30% mushroom patties were rated with the highest amount of firm texture, resulting in less acceptance; although the scores were still above the middle point of the scale. The results were consistent with the literature that the texture of beef patties with mushrooms substitution up to 30% was acceptable (Wong et al., 2019), while texture was even more preferred in high mushroom/low meat compared to low mushroom/high meat mixed dishes (Spencer & Guinard, 2018).

Overall acceptability would be associated with a multiple endogenous sensory modalities including acceptability and intensities of appearance, flavor, and texture. This study indicated that overall acceptability of mushroom–egg white patties was mainly affected by overall flavor liking, according to the PLS results (Figure 2). Our results are well aligned with the literature, suggesting that flavor acceptance is the most important sensory modality for overall acceptability (Andersen et al., 2019). Although overall flavor acceptance has been frequently used a few studies examining the acceptability of mushroom or mushroom products (Aisala et al., 2020; Spencer & Guinard, 2018; Wong et al., 2017, 2019), studies using statistical tools to correlate between overall acceptability and overall flavor acceptability are scarce.

Linking acceptability of sensory attributes to their intensities, a positive correlation between intensities of mushroom, meaty, and egg white flavors and their corresponding acceptance, respectively, and a negative correlation between the firmness acceptance and intensity were identified. It should be noted that a linear correlation might not exist. Practically, consumers prefer an optimal level of stimulus resulting highest acceptance response. The relationship between flavor intensity and acceptance response followed the inverted U‐shape, most likely (Tuorila, 1996). In another word, the ratio between ingredients to make a balanced and pleasant flavor is essential to create an appealing product.

Flavor pairing is a concept containing principles of congruent, harmonious, balanced intensities of tastes, flavor, texture, and aftertaste (Eschevins et al., 2019). Flavor pairing of mushroom–egg white has never been reported. Research related to flavor pairing has primarily been focused on food and beverage flavor pairing (Eschevins et al., 2019; Spence, 2020). This study indicated that consumer acceptance toward mushroom–egg white flavor pairing was positive, ranging from “either like or dislike” to “like moderately” of all mushroom–egg white patties. The 10% mushroom–egg white patties had the best flavor pairing between mushroom and egg white flavor, no matter the mushroom types and cooking methods. It further confirmed that the ratio of mushroom and egg white for the formula was an important factor influencing consumer acceptability.

Participant hedonic ratings of mushroom–egg white patties would be impacted by extragenous factors such as cooking method, mushroom type, and mushroom level. In this study, consumer likeability toward mushroom patties was significantly impacted by the cooking method. The cooking process results in a loss of moisture, formation of protein gel, and promotion of Maillard reaction, which create the aroma, flavor, and color commonly found in cooked foods. During the thermal process of *Agaricus bisporus* mushrooms, the total content of volatile compounds increased over three folds (Grosshauser & Schieberle, 2013; MacLeod & Panchasara, 1983). The number and concentration of pyrazines, furans, and pyrroles considerably increased during the drying of cepes and oyster mushrooms (Misharina et al., 2010). More volatiles were found in boiled soup than in microwaved soup, while the levels of free amino acids and 5′‐nucleotides were higher in the microwaved soup (Singh et al., 2003). These studies indicated that cooking mushrooms could increase the umami taste and “meat‐like” flavor, coinciding with our study that roasted mushroom patties had higher meaty notes.

It is well recognized that different mushroom species possess very diverse sensory properties, with odor descriptions ranged from fruity, nutty, seafood, to curry (Aisala et al., 2018; Siddiq et al., 2018). Consumer acceptance on the addition of white mushroom to replace a partial amount of beef, chicken, or pork from difference dishes has been examined (Guinard et al., 2016; Spencer & Guinard, 2018; Tom et al., 2018; Wong et al., 2017, 2019). Nevertheless, the comparison of white and crimini mushrooms in the same food base has not been previously reported.

In this study, the impact of mushroom levels on consumer acceptance was obvious, yet not extremely negative even up to 30% mushroom being added. Considering all sensory attributes, the addition of mushroom was acceptable up to 20%, with steamed method and crimini mushroom most preferred. The impact of mushroom proportions in dishes on consumer hedonic response was dependent on applied products, and the acceptable levels could be up to 45% (Wong et al., 2017). This study indicated that cooking methods, mushroom types, and levels would make the final product different in organoleptic properties, consequently affecting consumer acceptance.

## 5 CONCLUSIONS

A total of 380 consumers participated in the hedonic tests of the 16 developed mushroom–egg white patty prototypes for four overall impression and five attribute liking and intensities. The patties received positive hedonic scores for overall acceptance and the likability of overall flavor, mushroom flavor, meaty flavor, egg white flavor, overall texture and firmness, except overall appearance acceptance for the roasted mushroom patties. Overall acceptability was highly correlated with endogenous sensory attributes of the likability of overall flavor, followed by appearance and texture. Mushroom–egg white flavor pairing was scored positively across all patties, indicating a good pairing of the two basic food materials. Consumer hedonic levels toward mushroom–egg white patty prototypes were significantly impacted by cooking methods, mushroom types, and mushroom levels.

The research provided insight into consumer acceptance toward egg white partially replaced with mushrooms. Mushroom–egg white patties with high sensory appeal are a promising approach to implement the increase of mushroom consumption. The model mushroom–egg white patties provided useful references for the food industry selecting the most suitable mushroom types and levels to be processed and cooked. The understanding of mushroom–egg white flavor pairing paved the way for appropriateness of blend use. The findings of this study could be quite useful for the food industry in developing mushroom‐based products and identifying factors that influence consumers to consume the mushroom–egg white patties.

## DATA AVAILABILITY STATEMENT

Our data is available for review.

## CONFLICTS OF INTEREST

The authors declare that they do not have any conflict of interest.

## Supporting information

Demographic information and consumer segments
