# Supplementary material for: Consumer acceptance of egg white partially substituted with mushrooms and mushroom–egg white flavor pairing
Source: Food Sci Nutr. 2021 Jan 28;9(3):1410–21. doi: 10.1002/fsn3.2105 (PMC7958559; doi:10.1002/fsn3.2105)
Supplement: Supplementary file 1 — Demographic information and consumer segments [file FSN3-9-1410-s001.pdf]

Supporting information

Demographic information and consumer segments

The exit survey provided clarification and characterization of the consumer demographic information. Within the total of 380 volunteer participants in this study, it included 82.8% females and 17.2% males with the age distribution of 62.3% ≤25 years old, 21.3% 25-35 years old, and 16.3% >35 years old. The distribution of the gender and age was not equal, since the preselection was mainly based on mushroom consumption frequency and non-allergies to egg. The demographic information showed that 20.9% of participants consumed mushrooms several times a week, 43.1% consumed 1-4 times a month, and 36% reported consuming mushroom occasionally. Fresh mushrooms were the major format of consumption, selected by 62.3% participants, followed by canned mushrooms 4.2%, mushroom in burgers 13.0%, all the three 10%, and the rest selected N/A. Regarding how they prepared and ate mushrooms, 40.6% chose in salads, 77.0% for cooked-searing, 40.8% for cooked-fried, and 31.8% for baked. This finding was supportive that freshly cooking mushrooms were the major pattern as previously reported (Jiang, House, Kim, & Percival, 2017). Regarding the egg consumption frequency, 61.9% stated that they consumed eggs several times a week and 25.1% for 1-4 times per month, while only 13.0% consumed egg only occasionally. The results verified that egg is the one of the most widely consumed commodity (Bertechini, 2017). Participants were also asked how they defined their diet. Only 2.1% consumers were vegan, 5.9% were vegetarian, 30.1% were flexitarian, while 61.5% were none of above, which meant there was no limits for their dietary. The distribution of diet was consistent with a general population (Miki, Livingston, Karlsen, Folta, & McKeown, 2020). Overall, the participants recruited for this study represented a group who had high consumption frequency of both mushroom and egg.

Of all the aforementioned seven demographic variables, none of the categories showed significantly ($p \leq 0.05$) different and the effect sizes were small ($\eta^2 \leq 0.01$, data not shown). It implied that consumer preference toward mushroom patties was not determined by a single demographic factor and most likely by a multiple variances. Cluster analysis (Agglomerative hierarchical clustering, AHC) was then used to segment groups of consumers and find similar characters. Four consumer preference segments were identified based on all attributes. Cluster 1 had 94 participants (24.7%), cluster 2 had 100 (26.3%), cluster 3 had 120 (31.6%), and cluster 4 had 66 (17.4%), respectively. Preference of each of consumer cluster is shown in **Supplemental Figure**. Participants in cluster 1 preferred steamed mushroom patties, cluster 4 consumer preferred roasted mushroom patties, while cluster 2 and 3 consumers did not had preference on cooking method (**Supplemental Figure A**). Consumers in all four clusters did not show preference differences regarding mushroom type (**Supplemental Figure B**), however, there were differences on mushroom levels. Cluster 1 consumers exhibited preference on 10% mushroom patties, cluster 2 most preferred 20%, cluster 3 most preferred 30%, while respondents in cluster 4 liked control product most and 30% mushroom patties least (**Supplemental Figure C**). It implied that mushroom patties were favored by majority of participants (82.6%, the sum of clusters 1-3).

Cluster analysis of participants provided a deeper understanding of consumer preferences and revealed their behavior patterns. This study showed that one segment of consumers (Cluster 1) absolutely preferred steamed patties with 10% mushrooms, while another segment of consumers (Cluster 4) actually preferred the roasted patties without mushrooms. The other consumer segment (Cluster 2 and 3) did not show preference toward cooking method, but exhibited preference on high mushroom levels (20% and 30%). It was likely that each cluster reflected genuinely diverse personal preferences for mushroom levels and cooking methods. Consumer segments have been

analyzed and observed for preference on mushroom species and mushroom mixed dishes (Aisala et al., 2020; Spencer & Guinard, 2018). The consumer segments provided evidence for incorporating mushrooms in dishes that targeted individual groups of consumers and gave relevant information to different end users.

References

Aisala, H., Manninen, H., Laaksonen, T., Linderborg, K. M., Myoda, T., Hopia, A., & Sandell, M. (2020). *Linking volatile and non-volatile compounds to sensory profiles and consumer liking of wild edible nordic mushrooms* doi:https://doi-org.ezp.twu.edu/10.1016/j.foodchem.2019.125403

Bertechini, A. G. (2017). Economic and cultural aspects of the table egg as an edible commodity. *Egg innovations and strategies for improvements* (pp. 223-232) Elsevier Inc. doi:10.1016/B978-0-12-800879-9.00021-4

Jiang, Y., House, L. A., Kim, H., & Percival, S. S. (2017). Zero-inflated ordered probit approach to modeling mushroom consumption in the united states. *International Food and Agribusiness Management Review, 20*(5), 655-672. doi:10.22434/IFAMR2017.0006

Miki, A. J., Livingston, K. A., Karlsen, M. C., Folta, S. C., & McKeown, N. M. (2020). Using evidence mapping to examine motivations for following plant-based diets. *Current Developments in Nutrition, 4*(3) doi:10.1093/cdn/nzaa013

Spencer, M., & Guinard, J. -. (2018). The flexitarian flip™: Testing the modalities of flavor as sensory strategies to accomplish the shift from meat-centered to vegetable-forward mixed dishes. *Journal of Food Science, 83*(1), 175-187. doi:10.1111/1750-3841.13991

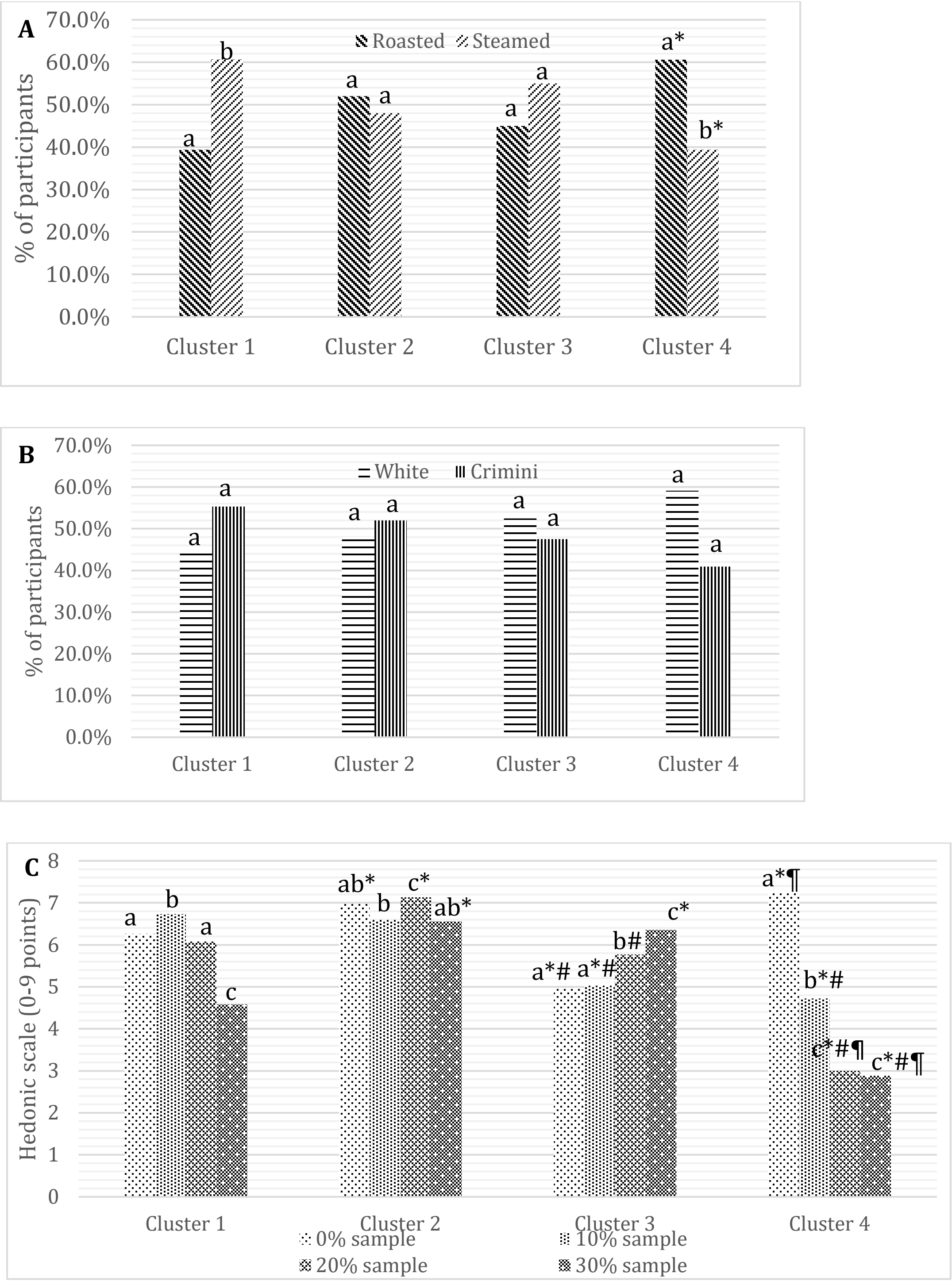

Supplemental Figure. Preference of each consumer segment (cluster) differentiated by cooking method (A), mushroom type (B), and overall liking (C). Demographic information hierarchical clustering analysis (HCA) resulted in four clusters with cluster 1 (n=94), cluster 2 (n=100), cluster 3 (n=120), and cluster 4 (n=66). Different letters between cooking method (A), mushroom type (B), and mushroom-egg white blends (C) within a cluster indicate significant differences between samples. Difference symbols across different clusters show significant difference between samples. $p<0.01$.
